# Computational guided identification of potential leads from *Acacia pennata* (L.) Willd. as inhibitors for cellular entry and viral replication of SARS-CoV-2

**DOI:** 10.1186/s43094-021-00348-7

**Published:** 2021-10-09

**Authors:** James H. Zothantluanga, Neelutpal Gogoi, Anshul Shakya, Dipak Chetia, H. Lalthanzara

**Affiliations:** 1 Department of Pharmaceutical Sciences, Faculty of Science and Engineering, Dibrugarh University, Dibrugarh, Assam 786004 India; 2 Department of Zoology, Pachhunga Univeristy College, Aizawl, Mizoram 796001 India

**Keywords:** SARS-CoV-2, COVID-19, Main protease, Furin, Molecular docking, Isovitexin, Apigenin-6-C-glucoside

## Abstract

**Background:**

Coronavirus disease 2019 (COVID-19) caused by severe acute respiratory syndrome coronavirus 2 (SARS-CoV-2) started in 2019 and is still an on-going pandemic. SARS-CoV-2 uses a human protease called furin to aid in cellular entry and its main protease (M^pro^) to achieve viral replication. By targeting these proteins, scientists are trying to identify phytoconstituents of medicinal plants as potential therapeutics for COVID-19. Therefore, our study was aimed to identify promising leads as potential inhibitors of SARS-CoV-2 M^pro^ and furin using the phytocompounds reported to be isolated from *Acacia pennata* (L.) Willd.

**Results:**

A total of 29 phytocompounds were reported to be isolated from *A. pennata*. Molecular docking simulation studies revealed 9 phytocompounds as having the top 5 binding affinities towards SARS-CoV-2 M^pro^ and furin. Among these phytocompounds, quercetin-3-*O*-*α*-L-rhamnopyranoside (C_18), kaempferol 3-*O*-*α*-L-rhamnopyranosyl-(1 → 4)-β-D-glucopyranoside (C_4), and isovitexin (C_5) have the highest drug score. However, C_18 and C_4 were not selected for further studies due to bioavailability issues and low synthetic accessibility. Based on binding affinity, molecular properties, drug-likeness, toxicity parameters, ligand interactions, bioavailability, synthetic accessibility, structure–activity relationship, and comparative analysis of our experimental findings with other studies, C_5 was identified as the most promising phytocompound. C_5 interacted with the active site residues of SARS-CoV-2 M^pro^ (GLU166, ARG188, GLN189) and furin (ASN295, ARG298, HIS364, THR365). Many phytocompounds that interacted with these amino acid residues were reported by other studies as potential inhibitors of SARS-CoV-2 M^pro^ and furin. The oxygen atom at position 18, the –OH group at position 19, and the 6-C-glucoside were identified as the pharmacophores in isovitexin (also known as apigenin-6-C-glucoside). Other in-silico studies reported apigenin as a potential inhibitor of SARS-CoV-2 M^pro^ and apigenin-o-7-glucuronide was reported to show stable conformation during MD simulations with SARS-CoV-2 M^pro^.

**Conclusion:**

The present study found isovitexin as the most promising phytocompound to potentially inhibit the cellular entry and viral replication of SARS-CoV-2. We also conclude that compounds having oxygen atom at position 18 (C-ring), –OH group at position 19 (A-ring), and 6-C-glucoside attached to the A-ring at position 3 on a C_6_–C_3_–C_6_ flavonoid scaffold could offer the best alternative to develop new leads against SARS-CoV-2.

## Background

The coronavirus disease 2019 (COVID-19) pandemic caused by the severe acute respiratory syndrome coronavirus 2 (SARS-CoV-2) started in December 2019 from Wuhan, Hubei Province of China [1]. To date, COVID-19 is still an on-going pandemic and as of 2nd February, 2021, it has affected more than 102 million people globally and has killed more than 2 million people [2]. The clinical manifestation of COVID-19 includes shortness of breath or difficulty in breathing, fever, cough, headache, body ache, fatigue, sore throat, loss of taste or smell, nausea, vomiting, congestion or runny nose, and diarrhea [3]. Since the initial outbreak, scientists and researchers are working to develop therapies against SARS-CoV-2 [1]. Although a specific drug for COVID-19 is still not available, studies to repurpose existing drugs for COVID-19 is underway [4].

With time, the pharmacological treatment options for COVID-19 had increased significantly [5]. However, problems like adverse effects, toxicity, or drug interactions seem to hinder the clinical utility of repurposed drugs for COVID-19 [4]. In the latest development, few vaccines have been authorized or approved for use against SARS-CoV-2 infection in humans [6]. Despite the breakthrough in vaccines, several deaths were reported among the recipients of those that receive the COVID-19 vaccinations [7]. Amidst the dilemma surrounding the available therapeutic options for COVID-19, phytotherapy may offer a safe and effective treatment against SARS-CoV-2 infection [8, 9]. Studies reveal that viral diseases were successfully treated with the bioactive compounds from medicinal plants [1, 10, 11]. Therefore, phytomedicines may be a promising prospect for COVID-19 therapy [1].

**Fig. 1:**
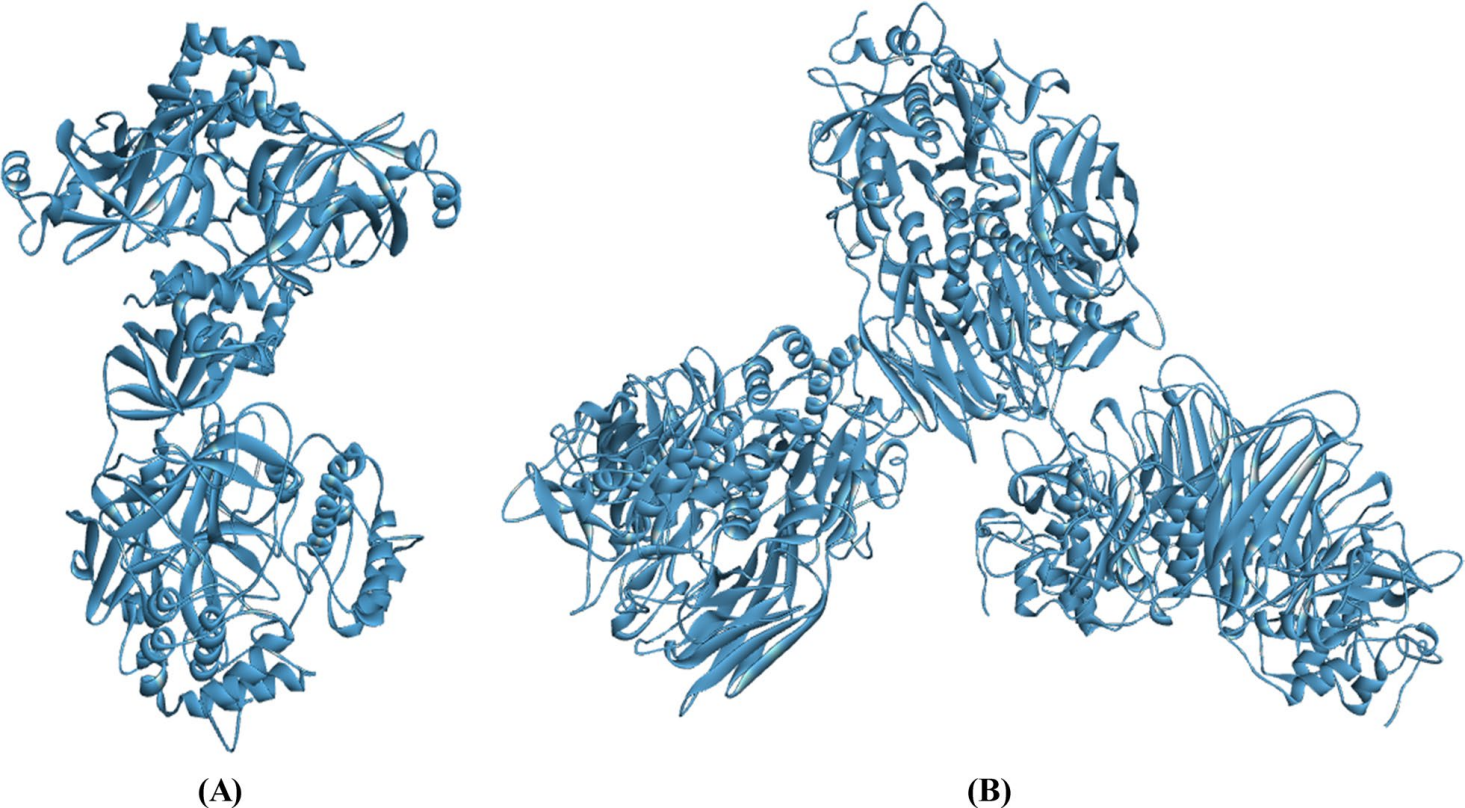
X-ray crystal structures of (**a**) SARS-CoV-2 M^pro^ and (**b**) furin

To develop an effective antiviral therapy, it is logical to validate the possible drug targets by identifying the primary proteins involved in a viral replication process. Proteolytic activation occurs when a human protease ‘furin’ cleaves the spike protein (SP) of SARS-CoV-2 [12, 13]. After the SP of SARS-CoV-2 is cleaved by furin, SARS-CoV-2 uses the S1 subunit of the spike protein (SP) to bind to the angiotensin-converting enzyme 2 and then the S2 subunit of the SP to fuse with the host cell to release its viral RNA [14]. After the viral RNA is released into the host cell, SARS-CoV-2 uses the host cell machinery to translate polyproteins from the RNA genome. Finally, cleavage of the polyproteins by the main protease (M^pro^) of SARS-CoV-2 results in replication and transcription of the viral genome [14–16]. The M^pro^ of SARS-CoV-2 is crucial for the replication of the virus [15]. Therefore, the M^pro^ of SARS-CoV-2 (Fig. 1) is recognized as the most favorable drug target SARS-CoV-2 [15, 17, 18]. Cleavage of SARS-CoV-2 SP by furin is necessary for proteolytic activation, viral fusion, and viral entry into the host cell [12, 13]. As inhibition of furin would prevent the entry of SARS-CoV-2 into the host cell, furin (Fig. 1) is also emerging as a favorable drug target in SARS-CoV-2 [12].

In the quest to find a safe and effective antiviral therapy for COVID-19, bioactive compounds of medicinal plants have gained the attention of researchers and scientists [8, 9, 19]. The endeavor to find possible leads from medicinal plants against SARS-CoV-2 has already started [1, 20, 21]. Interestingly, several phytochemicals of medicinal plants were reported to elicit an anti-viral activity [10, 11]. The potential of flavonoids as an antiviral agent against SARS-CoV-2 including other respiratory diseases has been reported [22, 23]. Moreover, the anti-inflammatory and antioxidant properties of flavonoids may also be beneficial to alleviate the severity of SARS-CoV-2 infection [24]. Therefore, in the search for an alternative anti-SARS-CoV-2 therapy, exploring a medicinal plant that is rich in flavonoids might be helpful.

*Acacia pennata* (L.) Willd. (Family: Mimosaceae) is an important Southeast Asian medicinal plant that is rich in flavonoids [25–27]. *A. pennata* is distributed in China, Thailand, India, Sri Lanka, Myanmar, Bhutan, and Bangladesh [25]. The plant parts of *Acacia* species like the leaves, barks, roots, pods, twigs, gum, and flowers are traditionally used to treat various health ailments [28]. The bark and the root bark of *A. pennata* are traditionally used to treat respiratory ailments like bronchitis and asthma [25, 28]. Headaches and fevers are also treated with *A. pennata* [25]. The stem bark of *A. pennata* is used as a traditional anti-inflammatory agent [29]. The bark of *A. pennata* is also traditionally used to treat gastrointestinal ailments like cholera and dysentery [25, 29]. Pharmacological activities like antiviral [30, 31], anti-inflammatory, antinociceptive [32], antioxidant [25], anti-parasitic [33], antimicrobial [34], and antidiabetic [35] had been scientifically investigated for *A. pennata*. The traditional utility of *A. pennata* to treat various health ailments may be attributed to the presence of flavonoids [26], terpenoids [27], phenols [28], and saponins [30] in the plant. Also, the young shoot tips of *A. pennata* is consumed as a vegetable as it is rich in nutrients and minerals [36–38].

*A. pennata* is widely available and is traditionally used to treat respiratory ailments. It is also used to treat other health ailments that are associated with COVID-19 such as fever, headache, inflammation, diarrhea like gastrointestinal issues such as dysentery, and cholera. The plant is also reported to possess anti-infective properties such as antiparasitic and antiviral activities. The facts provided above justifies the rationale to explore the phytocompounds of *A. pennata* for potential antiviral agents against SARS-CoV-2. Under urgent circumstances, computational approaches provide a great opportunity to identify natural compounds as potential inhibitors for cellular entry and viral replication of SARS-CoV-2. Therefore, the present study aims to identify potential leads against SARS-CoV-2 M^pro^ and furin using computational studies on the phytocompounds isolated from *A. pennata*.

## Methods

**Fig. 2:**
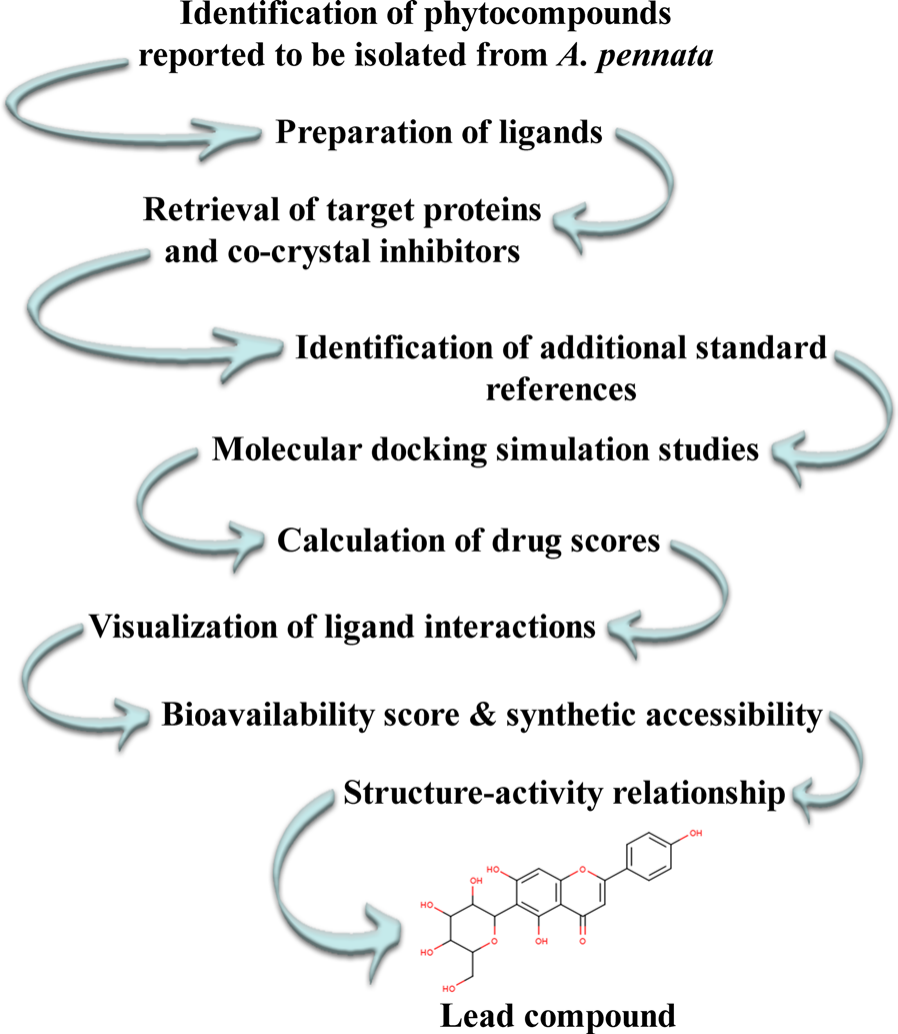
Research methodology of the present study

The research methodology of the current study is summarized in Fig. 2.

### Identification of phytocompounds reported to be isolated from *A. pennata*

An exhaustive online literature survey was conducted between November 2020 up to February 2021 to identify the phytocompounds that had been isolated from *A. pennata*. Academic databases like ‘PubMed’, ‘Science Direct’, and ‘Google Scholar’ were utilized for the literature survey. To search relevant papers, keywords such as ‘phytochemistry’, ‘phytoconstituents’, and ‘phytocompounds’ were used in combination with ‘*Acacia pennata*’. The phytocompounds that had been isolated from *A. pennata* were identified from the retrieved literature.

### Preparation of ligands

The ligands used in the study were prepared manually. The chemical structures of the isolated phytocompounds of *A. pennata* were sketched with MarvinSketch 20.10 software [39]. The correctness of the chemical structures was validated with the ‘Structure checker’ add-on that is available on the MarvinSketch 20.10 software. Then, the chemical structures were saved in MDL SDfile ‘^*^.sdf ^*^.sd’ format.

### Retrieval of target proteins and co-crystal inhibitors

The X-ray crystal structure of SARS-CoV-2 M^pro^ (PDB ID: 6M2N) was downloaded in ‘.pdb’ format from the RCSB-PDB website [40]. The co-crystal inhibitor (3WL) of SARS-CoV-2 M^pro^ was downloaded in ‘.sdf’ format from the RCSB-PDB website [41]. The X-ray crystal structure of human furin (PDB ID: 4RYD) was downloaded in ‘.pdb’ format from the RCSB-PDB website [42]. The co-crystal inhibitor (para-guanidinomethyl-Phac-R-Tle-R-Amba) of human furin was also downloaded in ‘.sdf’ format from the RCSB-PDB website [43]. The protein structures were pre-processed to remove water. The co-crystal inhibitors obtained along with the target proteins will be used as standard references.

### Identification of additional standard references

An anticancer drug ‘carmofur’ was reported to inhibit SARS-CoV-2 M^pro^ in Vero E6 cells (EC_50_ = 24.30 ± 3.61 µM) [44]. Another study reported that naphthofluorescein inhibited furin that is responsible for the cleavage of SARS-CoV-2 SP [12]. Owing to their experimental evidence against SARS-CoV-2 M^pro^ and furin, carmofur and naphthofluorescein will be used as a standard reference in addition to the co-crystal inhibitors. The chemical structures of carmofur and naphthofluorescein were prepared with MarvinSketch 20.10 software [39]. The correctness of their structures was checked using the ‘Structure checker’ add-on available on the MarvinSketch 20.10 software. Their structures were then saved in MDL SDfile ‘^*^.sdf ^*^.sd’ format.

### Molecular docking simulation studies

To predict the binding affinity between the target proteins and the phytocompounds, molecular docking simulation studies were carried out with Autodock Vina 1.1.2 on PyRx 0.8 virtual screening platform [45, 46]. In this study, chain A of SARS-CoV-2 M^pro^ and chain A of furin were used as the drug targets [40, 42]. According to the standard protocols, the three-dimensional affinity grid box in the Vina search space of PyRx 0.8 tool should enclose the entire protein for blind docking [47, 48]. However, the protein–co-crystal inhibitor complex was used to manually identify the three-dimensional coordinates of the active binding sites for SARS-CoV-2 M^pro^ and furin on the PyRx 0.8 tool. After the target protein was loaded onto the PyRx virtual screening platform, the target protein was expanded to reveal different chains that made up the protein. All the chains except chain A were removed from the scene for each protein. The protein-data bank format of the chain A of each protein was converted to ‘pdbqt.’ format wherein hydrogens were added during the conversion process. The sequence of the amino acids including the co-crystal inhibitor were revealed by expanding chain A. The atoms of the co-crystal inhibitor were labelled to identify the accurate location of the co-crystal inhibitor that is present at the active binding site of the protein. The centre of the 3D affinity grid box (*x* = 25.0 Å; *y* = 25.0 Å; *z* = 25.0 Å) and the co-crystal inhibitor were adjusted to align so that the affinity grid box was able to cover all the amino acid residues at the active binding site of the protein. Then, the active binding site coordinates of SARS-CoV-2 M^pro^ (*x* =  − 33.1066; *y* =  − 64.6263; *z* = 41.2995) and furin (*x* = 28.0166; *y* = 28.3695; *z* =  − 6.0437) were manually adjusted for each docking process to simulate the active binding sites of the co-crystal ligands. The rest of the parameters such as energy minimization for protein and ligands were kept default. The molecular docking simulation studies were carried out as per the standard protocols of PyRx software for the phytocompounds and the standard references [47]. The phytocompounds with the top 5 binding affinities towards the active binding site of SARS-CoV-2 M^pro^ and furin were selected for further analysis.

### Calculation of drug scores

The drug score of the standard references and the phytocompounds with the top 5 binding affinities for SARS-CoV-2 M^pro^ and furin were calculated using ORISIS Data Warrior v5.2.1 software [49]. The Data Warrior v.5.2.1 software takes several parameters (molecular properties, drug-likeness, and toxicity) into consideration to calculate the drug score. To select a phytocompound for further studies, it is important to make sure that the compound is safe and has good molecular properties. The Data Warrior v.5.2.1 software provides a low drug score for toxic compounds with undesirable molecular properties. Therefore, three phytocompounds with the highest drug scores were selected for further analysis.

### Visualization of ligand interactions

Even though a phytocompound is found to have a high binding affinity towards a target protein, it is important to make sure that the phytocompound interacts with the amino acid residues at the active binding site of the protein. Protein–ligand interactions like hydrogen bonding, hydrophobic interactions, electrostatic interactions, and the interacting active site residues of the proteins were visualized for both the standard references and the three phytocompounds with the highest drug scores. Discovery Studio Visualizer v20.1.0.19295 software was used to visualize the 2-dimensional ligand interactions. The 3-dimensional binding pose of the phytocompounds and the standard references towards the target proteins were visualized using PyMOL molecular graphics system, Version 2.4.1 Schrodinger, LLC. [50]. The ligand interactions of the standard references and the potential leads were comparatively analyzed.

### Bioavailability score and synthetic accessibility

A compound can be effective as a drug only if it is bioavailable [51]. Since bioavailability issues can slow down the process of drug development [52], preliminary investigation on the bioavailability of the phytocompounds is important. Synthetic accessibility is a fingerprint-based computational approach to determine the level of difficulty for synthesizing a compound [51]. As we aim to identify a potential lead from phytocompounds, it will be favourable for the identified lead to have high synthetic accessibility so that potent antiviral agents can be synthesized using the scaffold and pharmacophores of the lead compound. Therefore, after it was confirmed that multiple interactions occurred between the phytocompounds and the target protein, the bioavailability score and the synthetic accessibility of the phytocompounds were predicted with the SwissADME web tool [51].

### Structure–activity relationship

The structure–activity relationship of the phytocompound with the highest bioavailability score and the best synthetic accessibility was analyzed. A mapped structure of the phytocompound was prepared with MarvinSketch 20.10 software. The functional groups, chains, or atoms of the structure of the phytocompound that interacted with the amino acid residues at the active binding site were manually identified. The observations made in the study were compared with several other published data.

## Results

### Phytocompounds reported to be isolated from *A. pennata*

**Fig. 3:**
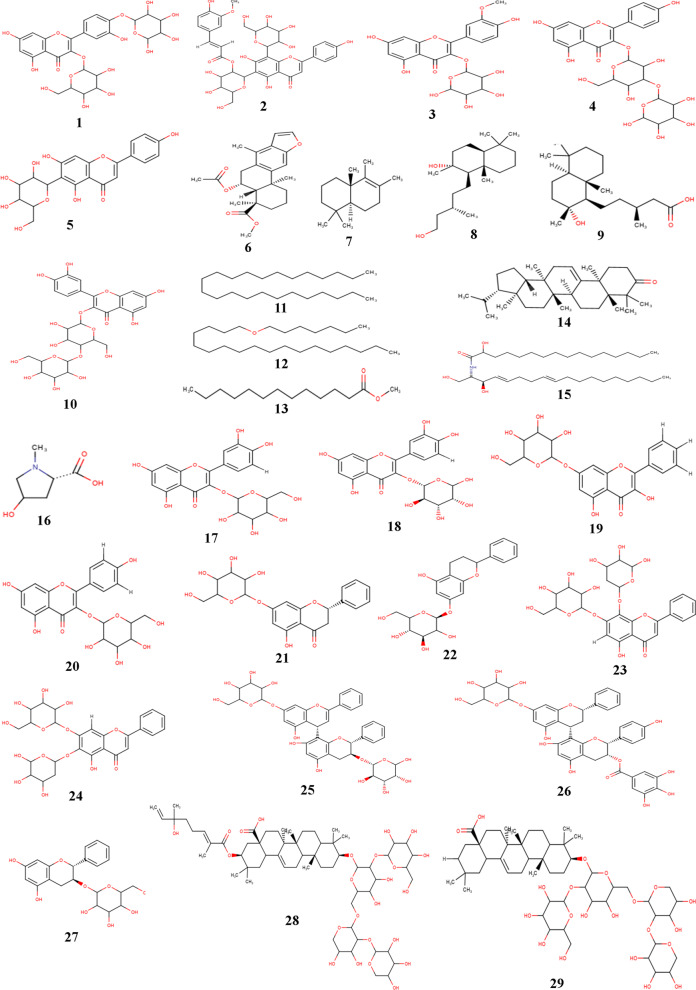
Chemical structures of phytocompounds reported to be isolated from *A. pennata*

The list of phytocompounds that had been reported to be isolated from *A. pennata* is given in Table 1. The chemical structures of all the isolated phytocompounds are also presented in Fig. 3.

**Table 1:** List of phytocompounds isolated from *A. pennata*

Sl.No	Phytocompounds	Compound ID	Chemical class	Isolated from	References
1	Quercetin 4’-*O*-α-L-rhamnopyranosyl-3-*O*-β-D-allopyranoside	C_1	Flavonoid	Leaves	[32]
2	Apigenin 6-*C*-[2″-*O*-(E)-feruloyl-β-D-glucopyranosyl]-8-*C*-β-glucopyranoside	C_2	Flavonoid	Leaves	[32]
3	Isorhamnetin 3-*O*-α-L-rhamnopyranoside	C_3	Flavonoid	Leaves	[32]
4	Kaempferol 3-*O*-α-L-rhamnopyranosyl-(1 → 4)-β-D-glucopyranoside	C_4	Flavonoid	Leaves	[32]
5	Isovitexin	C_5	Flavonoid	Leaves	[32]
6	Taepeenin D	C_6	Terpenoid	Leaves	[27]
7	( +)-drim-8-ene	C_7	Terpenoid	Leaves	[27]
8	8,15-labdanediol	C_8	Terpenoid	Leaves	[27]
9	Labdanolic acid	C_9	Terpenoid	Leaves	[27]
10	Quercetin 3-*O*-β-D-glucopyranosyl-4-*O*-β- D-glucopyranoside	C_10	Flavonoid	Leaves	[27]
11	Tetracosane	C_11	Alkane	Twigs	[53]
12	1-(heptyloxy)-octadecane	C_12	Alkane	Twigs	[53]
13	Methyl tridecanoate	C_13	Ester	Twigs	[53]
14	Arborinone	C_14	Terpenoid	Twigs	[53]
15	Confertamide A	C_15	-	Twigs	[53]
16	4-hydroxy-1-methyl-pyrrolidin-2-carboxylic acid	C_16	Alkaloid	Twigs	[53]
17	Quercetin-3-*O*-β-D-glucopyranoside	C_17	Flavonoid	Aerial parts	[26]
18	Quercetin-3-*O*-α-L-rhamnopyranoside	C_18	Flavonoid	Aerial parts	[26]
19	Chrysin-7-*O*-β-D-glucopyranoside	C_19	Flavonoid	Aerial parts	[26]
20	Kaempferol 3-*O*-α-L-rhamnopyranoside	C_20	Flavonoid	Aerial parts	[26]
21	Pinocembrin-7-*O*-β-D-glucopyranoside	C_21	Flavonoid	Aerial parts	[26]
22	Koaburanin	C_22	Flavonoid	Aerial parts	[26]
23	5,7-dihydroxyflavone 7-*O*-β-D-glucopyranosyl-8-C-β-boivinopyranoside	C_23	Flavonoid	Aerial parts	[26]
24	5,7-dihydroxyflavone 6-C-β-boivinopyranosyl-7-*O*-β-D-glucopyranoside	C_24	Flavonoid	Aerial parts	[26]
25	(2R)-4’,7-dihydroxyflavan-(4a → 8)-(2R,3S)-3,5,7-trihdyroxyflavan-3″-*O*-α-L-rhamnopyranoside	C_25	Flavonoid	Aerial parts	[26]
26	(2S)-5,7-dihydroxyflavan-7-*O*-β-D-glucopyranoside-(4a → 8)-epiafzelechin-3-*O*-gallate	C_26	Flavonoid	Aerial parts	[26]
27	(2R,3S)-3,5,7-trihdyroxyflavan-3-*O*-α-L-rhamnopyranoside	C_27	Flavonoid	Aerial parts	[26]
28	21β-*O*-[(2*E*)-6-hydroxyl-2,6-dimethyl-2,7-octadienoyl] pitheduloside G	C_28	Saponin	Stem	[30]
29	Pitheduloside G	C_29	Saponin	Stem	[30]

### Molecular docking simulation studies

The binding affinities of the phytocompounds and the standard references (co-crystal inhibitors and additional standard references) towards SARS-CoV-2 M^pro^ and furin are given in Table 2.

**Table 2:** Binding affinities (kcal/mol) of the phytocompounds towards SARS-CoV-2 M^pro^ and furin

Sl. No	Compound ID	SARS-CoV-2 M^pro^	Furin
1	C_1	− 7.3	− 8.2
2	C_2	− 6	− 6.5
3	C_3	− 6.6	− 7.6
4	C_4	− 7.2	− 8
5	C_5	− 7.3	− 8
6	C_6	− 6	− 6.6
7	C_7	− 5.4	− 5.5
8	C_8	− 5	− 5.6
9	C_9	− 6	− 6.3
10	C_10	− 6.4	− 7.1
11	C_11	− 3.6	− 4.2
12	C_12	− 3.9	− 4.3
13	C_13	− 3.9	− 3.8
14	C_14	− 6.6	− 8.1
15	C_15	− 4.9	− 5.1
16	C_16	− 4.6	− 4.8
17	C_17	− 6.5	− 7.8
18	C_18	− 7.6	− 2
19	C_19	− 7	− 7.9
20	C_20	− 6.9	− 7.5
21	C_21	− 7.2	− 7.9
22	C_22	− 6.7	− 7.6
23	C_23	− 7.3	− 8.1
24	C_24	− 7.3	− 7.9
25	C_25	− 7.7	− 7.7
26	C_26	− 8	− 8.2
27	C_27	− 1.7	− 2.3
28	C_28	− 7	− 8.3
29	C_29	− 6.7	− 9
30	3WL	− 6.7	-
31	Carmofur	− 5.6	-
32	Para-guanidinomethyl-Phac-R-Tle-R-Amba	–	− 7
33	Naphthofluorescein	–	− 10

### Drug score

The molecular properties (molecular weight and lipophilicity), drug-likeness, toxicity (mutagenicity, tumorigenic, reproductive effective, and irritant), and the overall drug score of the standard references and the phytocompounds with the top 5 binding affinities towards SARS-CoV-2 M^pro^ and furin are given in Tables 3 and 4 respectively.

**Table 3:** Molecular properties, drug-likeness, toxicity parameters, and the overall drug score of phytocompounds with the top 5 binding affinities and the standard references towards SARS-CoV-2 M^pro^

Sl. No	Compound ID	Mol wt	cLogP	H-A	H–D	DL	MG	TG	RE	IT	Drug score
1	C_26	828.774	3.0928	17	11	− 3.9418	None	None	High	None	0.1106131
2	C_25	806.767	1.1416	17	11	− 3.7831	None	None	None	None	0.2135499
3	C_18	450.351	− 0.0954	12	8	0.32236	None	None	None	None	0.6301938
4	C_1	626.518	− 1.89	17	11	− 3.6941	None	None	None	None	0.2942673
5	C_5	432.380	− 0.078	10	7	− 1.9977	None	None	None	None	0.4577336
6	C_23	564.494	− 0.5393	14	8	− 2.2846	High	High	None	None	0.1216019
7	C_24	564.494	− 0.5393	14	8	− 2.2846	None	None	None	None	0.3377829
8	C_4	596.492	− 1.5868	16	10	0.38943	None	None	None	None	0.4808296
9	C_21	418.397	0.5121	9	5	− 3.7146	None	None	None	None	0.4171902
10	3WL	270.239	2.3357	5	3	0.28194	None	None	None	None	0.6981475
11	Carmofur	257.264	1.4226	6	2	− 13.647	High	None	High	None	0.16024

Mol wt. = Molecular weight; cLogP = partition coefficient between n−octanol and water; H−A = Hydrogen acceptor; H–D = Hydrogen donor; DL = Druglikeness; MG = Mutagenicity; TG = Tumorigenic; RE = Reproductive effective; IT = Irritant

**Table 4:** Molecular properties, drug-likeness, toxicity parameters, and the overall drug score of phytocompounds with the top 5 binding affinities and the standard references towards furin

Sl. No	Compound ID	Mol wt	cLogP	H-A	H–D	DL	MG	TG	RE	IT	Drug score
1	C_29	1045.22	− 0.0801	21	12	− 11.577	None	None	None	None	0.181405
2	C_28	1227.44	1.8063	24	13	− 16.06	None	None	None	High	0.088529
3	C_1	626.518	− 1.89	17	11	− 3.6941	None	None	None	None	0.2942673
4	C_26	828.774	3.0928	17	11	− 3.9418	None	None	High	None	0.1106131
5	C_14	438.737	7.815	1	0	− 6.3613	None	None	None	None	0.1257381
6	C_23	564.494	− 0.5393	14	8	− 2.2846	High	High	None	None	0.1216019
7	C_4	596.492	− 1.5868	16	10	0.38943	None	None	None	None	0.4808296
8	C_5	432.380	− 0.078	10	7	− 1.9977	None	None	None	None	0.4577336
9	C_18	450.351	− 0.0954	12	8	0.32236	None	None	None	None	0.6301938
10	PGPRTRA	763.950	− 2.896	19	15	− 4.1097	None	None	None	None	0.255768
11	Naphthofluorescein	432.430	6.1173	5	2	− 1.2682	Low	High	None	None	0.0831259

Mol wt. = Molecular weight; cLogP = partition coefficient between n−octanol and water; H−A = Hydrogen acceptor; H–D = Hydrogen donor; DL = Druglikeness; MG = Mutagenicity; TG = Tumorigenic; RE = Reproductive effective; IT = Irritant

### Ligand interactions

**Fig. 4:**
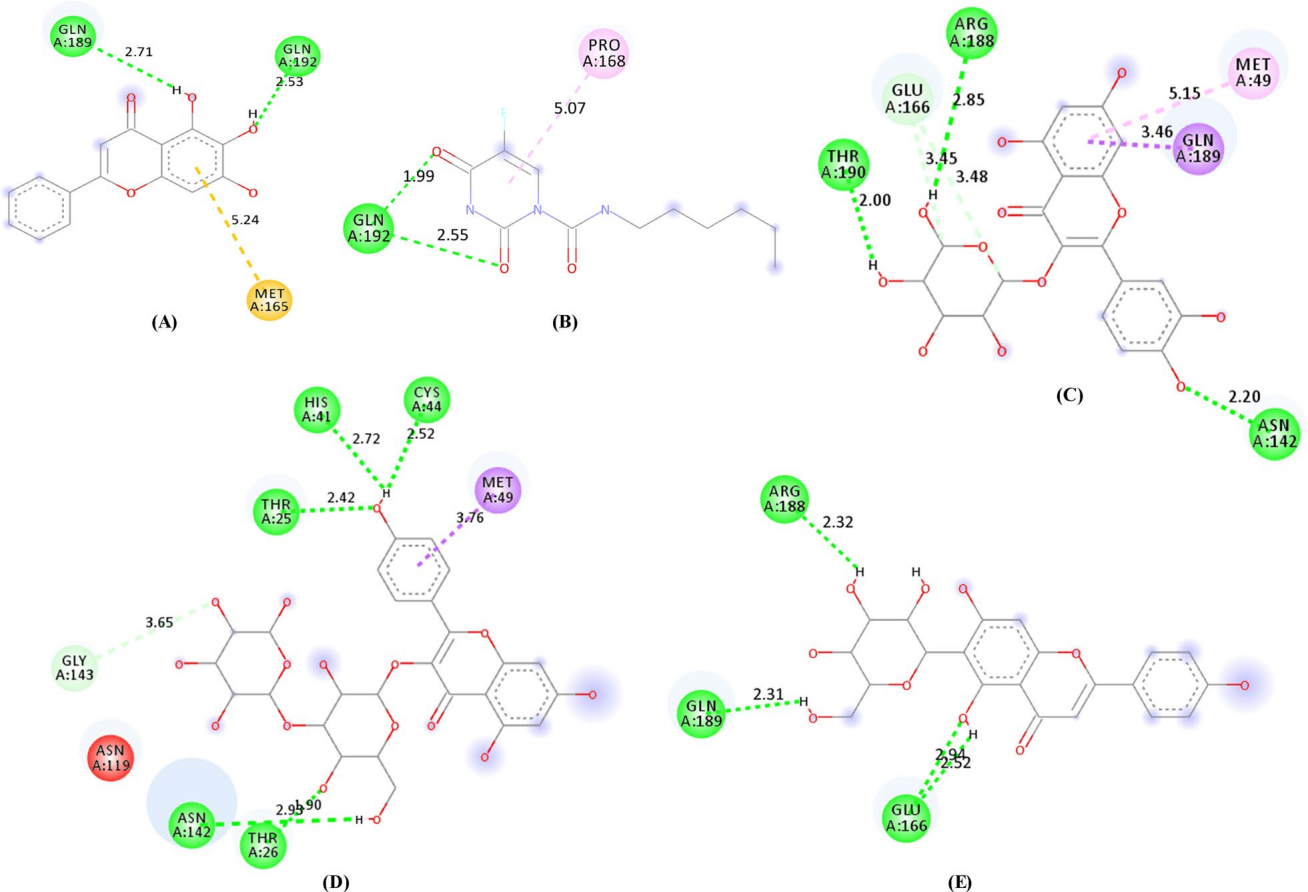
Visualization of 2-dimensional ligand interactions between the amino acid residues of the active binding pockets of SARS-CoV-2 M^pro^ with (**a**) 3WL, (**b**) carmofur, (**c**) C_18, (**d**) C_4 and (**e**) C_5

**Fig. 5:**
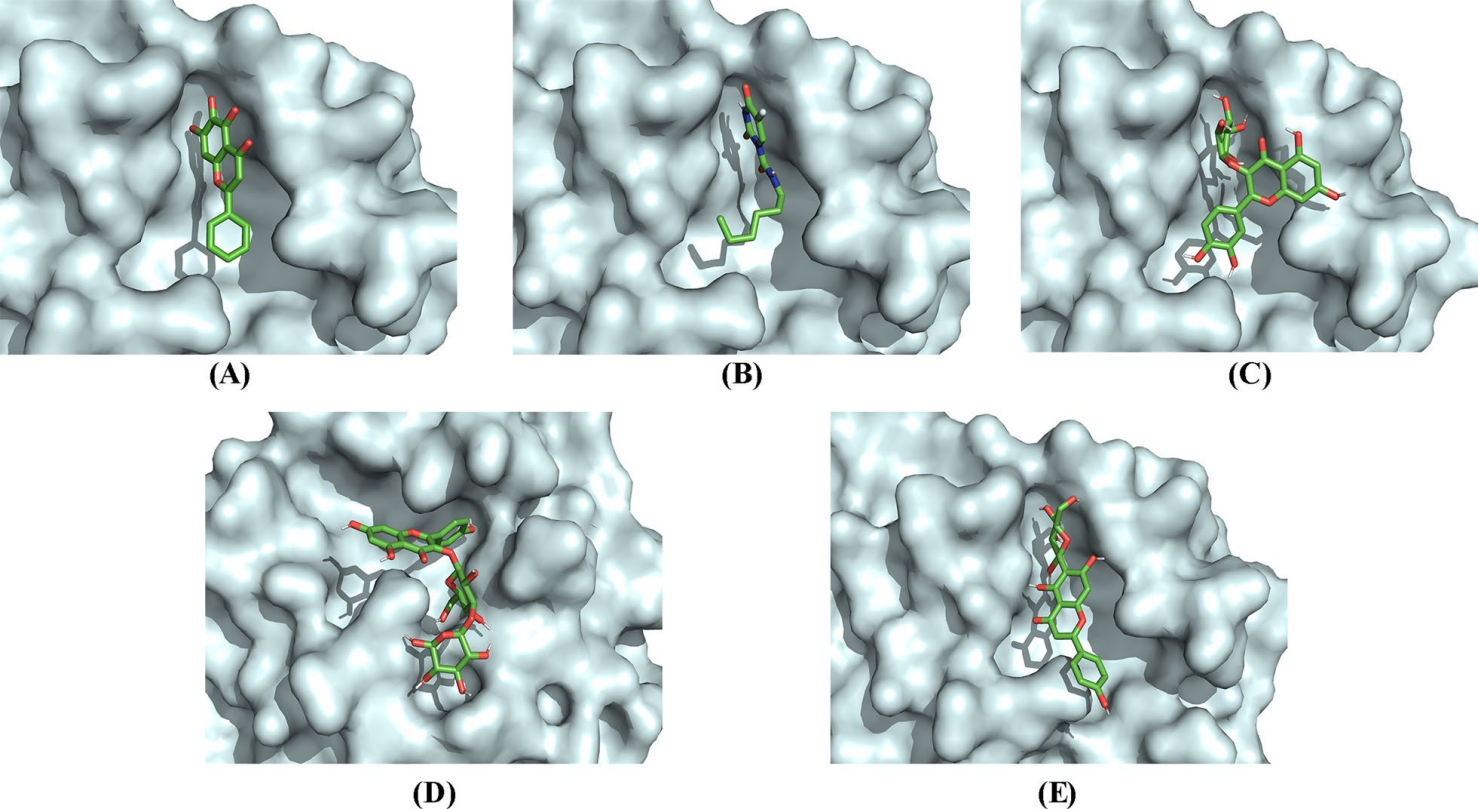
3-dimensional view at the biding pose of (**a**) 3WL, (**b**) carmofur, (**c**) C_18, (**d**) C_4 and (**e**) C_5 at the active binding pockets of SARS-CoV-2 M^pro^

Ligand interactions of the three phytocompounds with the highest drug scores (C_18, C_4, and C_5) and the standard references (3WL and carmofur) with the amino acid residues at the active binding site of SARS-CoV-2 M^pro^ in 2-dimensional view is given in Fig. 4. The 3-dimensional binding pose of the phytocompounds and the standard references at the active binding pockets of SARS-CoV-2 M^pro^ is given in Fig. 5. The binding parameters of the standard references and the phytocompounds with the amino acid residues at the active binding site of SARS-CoV-2 M^pro^ are given in Table 5.

**Table 5:** Binding parameters of the phytocompounds and the standard references with the amino acid residues at the active binding site of SARS-CoV-2 M^pro^

Sl. No	Compound ID	Conventional hydrogen bonding	Hydrophobic interaction	Carbon-hydrogen interaction	Electrostatic interaction
1	C_18	THR190, ARG188, ASN142	MET49, GLN189	GLU166	–
2	C_4	THR25, HIS41, CYS44, ASN142, THR26	MET49	GLY143	–
3	C_5	ARG188, GLN189, GLU166	–	–	–
4	3WL	GLN189, GLN192	–	–	MET165
5	Carmofur	GLN192	PRO168	–	–

**Fig. 6:**
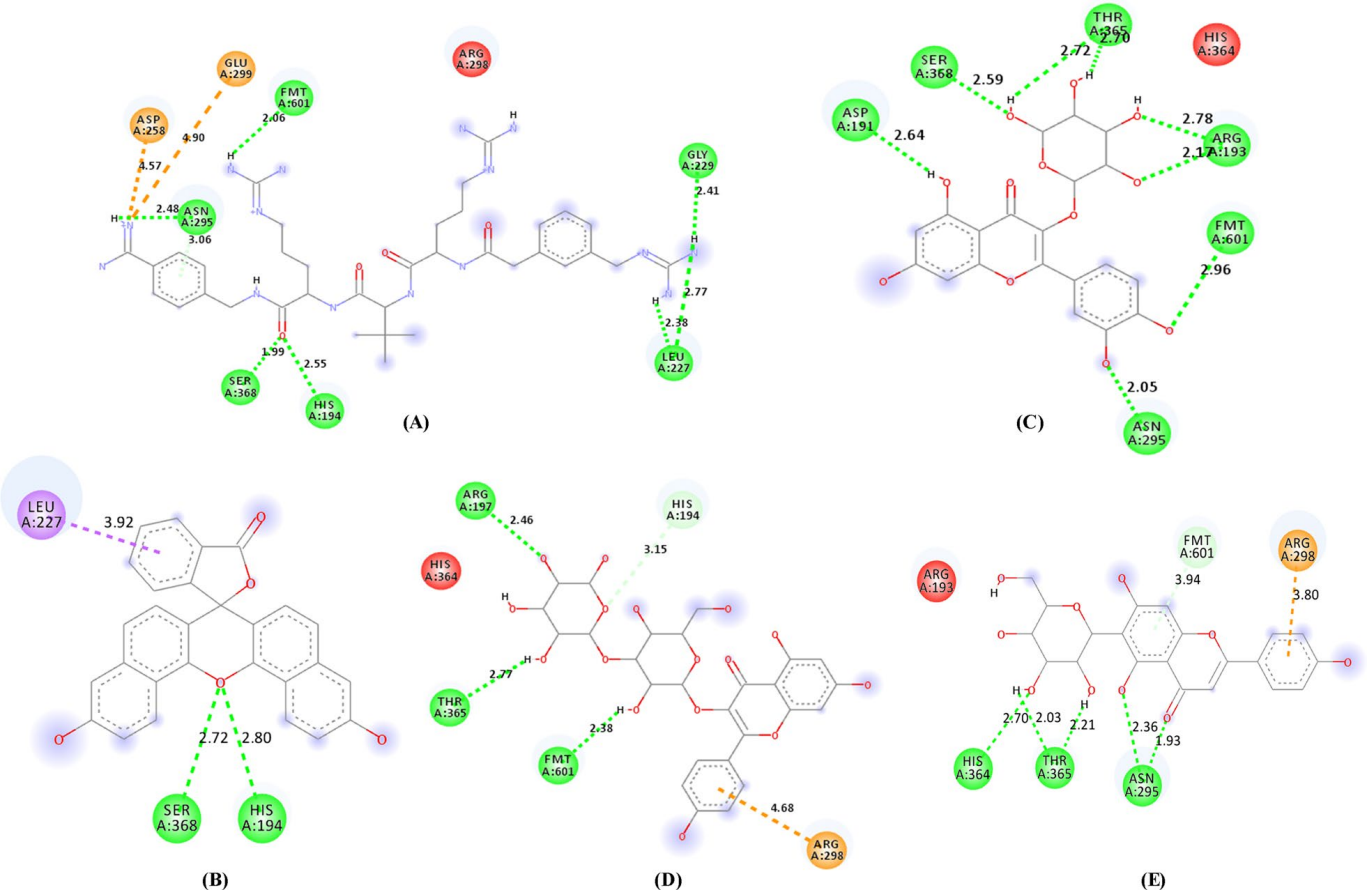
Visualization of ligand interactions between the amino acid residues of the active binding pockets of furin with PGPRTRA (**a**), naphthofluorescein (**b**), C_18 (**c**), C_4 (**d**) and C_5 (**e**)

**Fig. 7:**
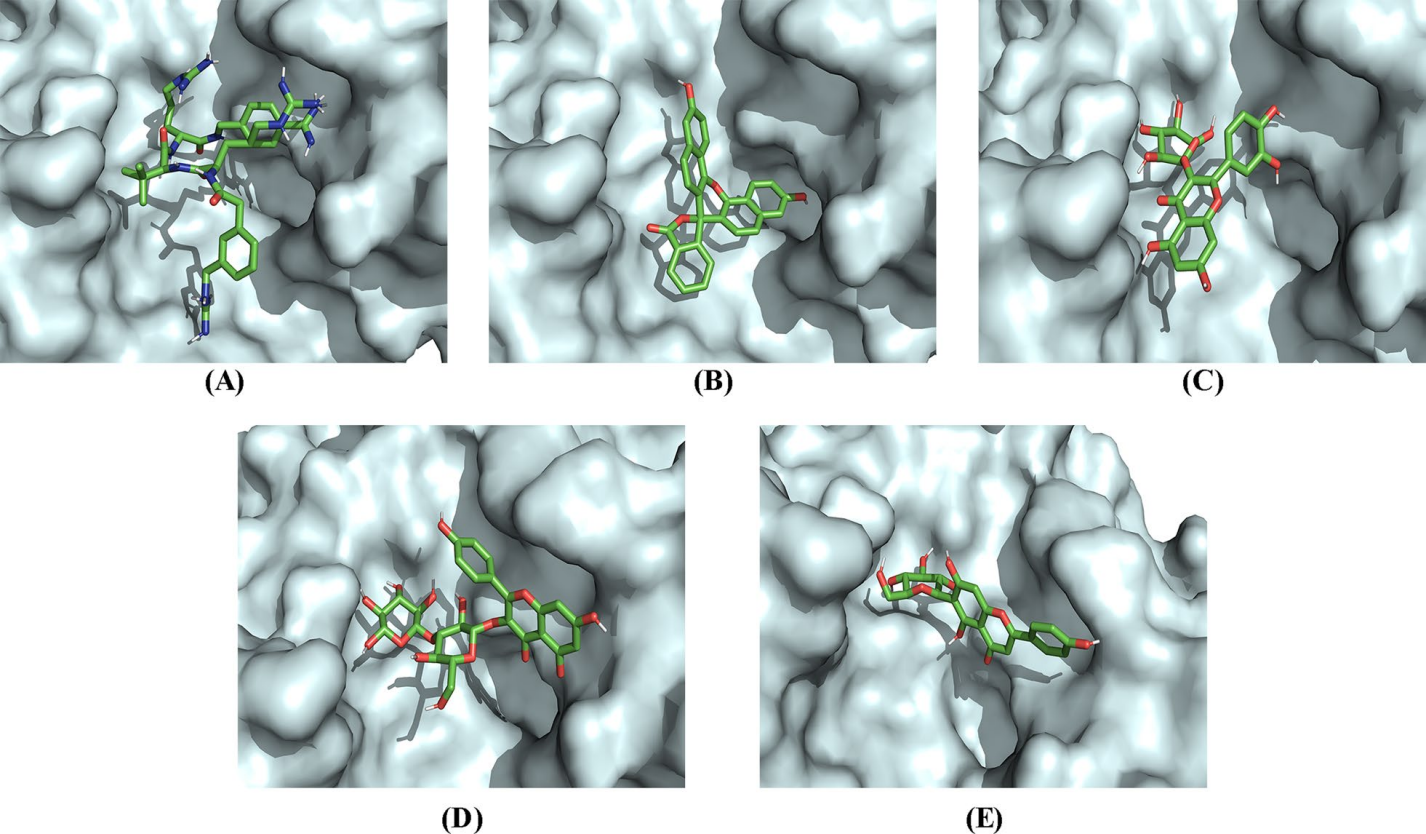
3-dimensional view at the biding pose of (**a**) PGPRTRA, (**b**) Naphthofluorescein, (**c**) C_18, (**d**) C_4 and (**e**) C_5 at the active binding pockets of furin

Ligand interactions of the three phytocompounds with the highest drug scores (C_18, C_4, and C_5) and the standard references (PGPRTRA and naphthofluorescein) with the amino acid residues at the active binding site of furin in 2-dimensional view is given in Fig. 6. The 3-dimensional binding pose of the phytocompounds and the standard references at the active binding pockets of furin is given in Fig. 7. The binding parameters of the standard references and the phytocompounds with the active binding site amino acid residues of furin are given in Table 6.

**Table 6:** Binding parameters of the phytocompounds and the standard references with the amino acid residues at the active binding site of furin

Sl. No	Compound ID	Conventional hydrogen bonding	Hydrophobic interaction	Carbon-hydrogen interaction	Electrostatic interaction
1	C_18	ASP191, SER368, THR365, ARG193, FMT601, ASN295	–	–	–
2	C_4	ARG197, THR365, FMT601		HIS194	ARG298
3	C_5	HIS364, THR365, ASN295	–	FMT601	ARG298
4	PGPRTRA	ASN295, FMT601, SER368, HIS194, LEU227, GLY229	–	–	ASP258, GLU299
5	Naphthofluorescein	HIS194, SER368	LEU227	–	–

### Bioavailability score and synthetic accessibility

The bioavailability score and synthetic accessibility of the three phytocompounds with the highest drug scores are given in Table 7. A compound should have a high bioavailability score with a low numerical value for its synthetic accessibility (1 = easy to synthesize; 10 = difficult to synthesize) [51].

**Table 7:** Bioavailability score and synthetic accessibility of the phytocompounds

Sl. No	Compound ID	Bioavailability score	Synthetic accessibility
1	C_4	0.17	6.35
2	C_5	0.55	4.99
3	C_18	0.17	5.19

### Structure–activity relationship of C_5

**Fig. 8:**
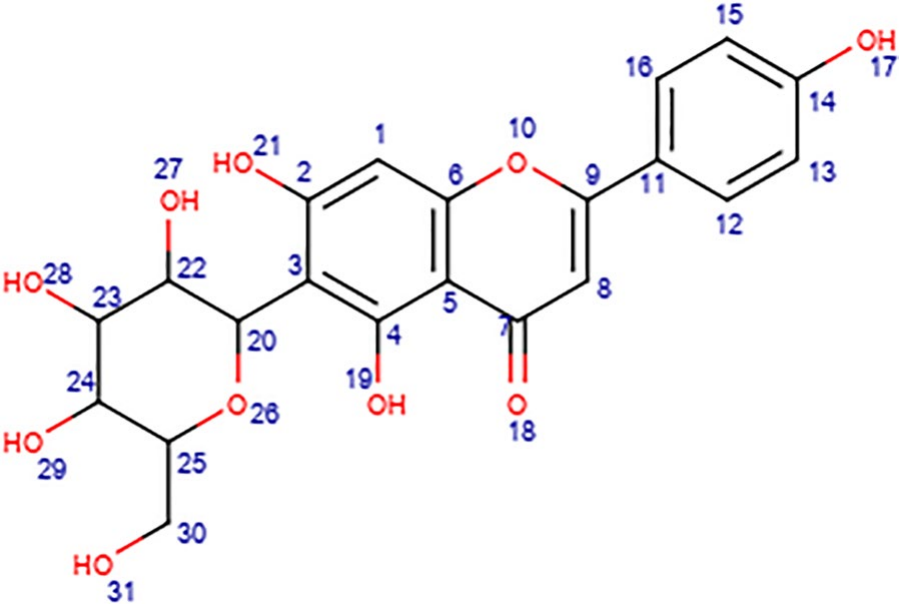
Mapped structure of C_5

The mapped structure of the phytocompound (C_5) with the highest bioactivity score and the best synthetic accessibility is given in Fig. 8.

The hydroxyl (–OH) group at position 19 attached to the A-ring of the C_6_-C_3_-C_6_ scaffold interacts with SARS-COV-2 M^pro^ by forming two conventional hydrogen bonds with GLU166 at the active binding site of the protein (Figs. 4 and 8). The –OH group at positions 28 and 31 of the 6-C glucoside attached to the A-ring of the C_6_-C_3_-C_6_ scaffold interacts with SARS-CoV-2 M^pro^ by forming conventional hydrogen bonds with ARG188 and GLN189 at the active binding site of the protein respectively (Figs. 4 and 8).

The –OH group at position 19 of the A-ring and the oxygen atom at position 18 of the C-ring interacts with furin by forming a conventional hydrogen bond with ASN295 at the active binding site of the protein (Figs. 6 and 8). The –OH group at positions 27 and 28 of the 6-C glucoside attached to the A-ring also interacts with furin by forming a conventional hydrogen bond with THR365 (Figs. 6 and 8). The –OH group at position 28 of the 6-C glucoside also formed a conventional hydrogen bond with HIS364 of furin. The B-ring of the C_6_-C_3_-C_6_ scaffold formed electrostatic interaction with ARG298 of furin (Figs. 6 and 8).

## Discussion

### Phytocompounds reported to be isolated from *A. pennata*

Literature survey reveals that 29 phytocompounds belonging to different classes of secondary metabolites like flavonoids, alkaloids, terpenoids, and saponins had been isolated from the stem, aerial parts, twigs, and leaves *A. pennata* (Table 1, Fig. 3). Recently, a team of researchers found that the hydromethanolic extract of *A. pennata* offers protection against DNA damage induced by acetaminophen in rat liver and they had also detected 5 new flavonoid-glycosides (Apigenin 6,8-di-C-hexoside, Apigenin-6-C-pentoside-8-C-hexoside, Apigenin-6-C-hexoside-8-C-pentoside, Kaempferol 3,7-di-O-hexoside, and Luteolin-6-C-pentoside-8-C-hexoside) in the hydromethanolic extract [54]. However, these newly detected phytocompounds were not included in the study as there is still no report on their isolation. Another recent study reported that phosphodiesterase-5 was inhibited by the ethanolic extract of the leaves of *A. pennata* [55]. The same study highlighted four phytocompounds (quercetin-3-*O*-β-D-glucopyranoside, chrysin-7-*O*-β-D-glucopyranoside, koaburanin, and pinocembrin-7-*O*-β-D-glucopyranoside) as identified phytochemicals in the ethanolic extract [55]. The isolation of these four phytocompounds had been reported and are included in the present study. The majority of the isolated phytocompounds (*n* = 17) from *A. pennata* belonged to the class of flavonoids. The antiviral activity of flavonoids is well explored [56, 57] and their potential role against coronavirus infection had also been thoroughly discussed [24, 58]. Since *A. pennata* is rich in flavonoids, the chances of finding a potential lead from *A. pennata* as a SARS-CoV-2 inhibitor also increases. The study was initially proposed to limit the virtual screening to isolated flavonoids only. However, a literature survey revealed that a saponin ‘21β-*O*-[(2*E*)-6-hydroxyl-2,6-dimethyl-2,7-octadienoyl] pitheduloside G’ isolated from *A. pennata* exhibited an antiviral activity [30]. Therefore, in addition to flavonoids, the chances to find a potential lead from other classes of secondary metabolites also exist. Thus, irrespective of their chemical class, it was decided to screen all the reported phytocompounds that had been isolated from *A. pennata*.

### Molecular docking simulation studies

The molecular docking simulation experiment revealed the binding affinities of the phytocompounds and the standard references (co-crystal inhibitors and additional standard references) towards SARS-CoV-2 M^pro^ and furin (Table 2). A high binding affinity signifies that a ligand will bind to the target protein with minimum energy [1]. Thus, a ligand with a high binding affinity towards the target protein is often desirable to be selected for further studies. In general, the binding affinities of the phytocompounds towards SARS-CoV-2 M^pro^ ranged from − 8.0 to − 1.7 kcal/mol while the binding affinities of the phytocompounds towards furin ranged from − 9.0 to − 2.0 kcal/mol. When a molecular docking simulation study was conducted on a PyRx 0.8 software, a total of 9 poses were generated for each ligand [47]. The PyRx 0.8 automatically selects the first pose with the highest binding affinity as the best pose.

Phytocompounds with the top 5 binding affinities towards SARS-CoV-2 M^pro^ are C_26 (− 8.0 kcal/mol), C_25 (− 7.7 kcal/mol), C_18 (− 7.6 kcal/mol), C_1 (− 7.3 kcal/mol), C_5 (− 7.3 kcal/mol), C_23 (− 7.3 kcal/mol), C_24 (− 7.3 kcal/mol), C_4 (− 7.2 kcal/mol) and C_21 (− 7.2 kcal/mol) (Table 2). The co-crystal inhibitor ‘3WL’ and the additional standard reference ‘Carmofur’ showed a binding affinity of − 6.7 kcal/mol and − 5.6 kcal/mol respectively (Table 2). The phytocompounds with the top 5 binding affinities (C_26, C_25, C_18, C_1, C_5, C_23, C_24, C_4, and C_21) showed a better binding affinity towards SARS-CoV-2 M^pro^ than the standard references (3WL and carmofur).

Phytocompounds with the top 5 binding affinities towards furin are C_29 (− 9.0 kcal/mol), C_28 (− 8.3 kcal/mol), C_1 (− 8.2 kcal/mol), C_26 (− 8.2 kcal/mol), C_14 (− 8.1 kcal/mol), C_23 (− 8.1 kcal/mol), C_4 (− 8.0 kcal/mol), C_5 (− 8.0 kcal/mol) and C_18 (− 8.0 kcal/mol) (Table 2). The co-crystal inhibitor ‘Para-guanidinomethyl-Phac-R-Tle-R-Amba’ (PGPRTRA) and the additional standard reference ‘Naphthofluorescein’ showed a binding affinity of − 7.0 kcal/mol and − 10.0 kcal/mol respectively (Table 2). The phytocompounds (C_29, C_28, C_1, C_26, C_14, C_23, C_4, C_5, and C_18) showed a better binding affinity towards furin than the co-crystal inhibitor (PGPRTRA) but not a better binding affinity than the additional standard reference (Naphthofluorescein).

### Drug score of phytocompounds with the top 5 binding affinities

The molecular properties, drug-likeness, toxicity parameters, and the overall drug score of the standard references and the phytocompounds with the top 5 binding affinities towards SARS-CoV-2 M^pro^ and furin are given in Tables 3 and 4 respectively. The ORISIS Data Warrior v5.2.1 software uses molecular properties like molecular weight, cLogP, hydrogen acceptors, hydrogen donors, drug-likeness, and toxicity parameters like mutagenicity, tumorigenic, reproductive effective, and irritant to calculate the overall drug score of a compound. Toxicity is one of the reasons why many drugs had to be withdrawn from the market [59]. In addition to the binding affinity, the drug score can be used as a filter to narrow down and select suitable compounds for further studies. Thus, the drug score provides a referential value that can be used to judge the overall potential of a compound to qualify as a drug [60]. The reference standards against SARS-CoV-2 M^pro^ had a drug score of 0.6981475 (3WL) and 0.16024 (Carmofur) while the reference standards against furin had a drug score of 0.255768 (PGPRTRA) and 0.0831259 (Naphthofluorescein). Among the phytocompounds with the top 5 binding affinities towards SARS-CoV-2 M^pro^ and furin, C_18 (0.6301938), C_4 (0.4808296) and C_5 (0.4577336) had the highest drug scores (Tables 3 and 4). The drug score of all the three phytocompounds is higher than all the standard references except for the co-crystal inhibitor of SARS-CoV-2 M^pro^ (3WL). As C_18, C_4 and C_5 were free from all possible signs of toxicity, they were subjected to further analysis to check whether they had interacted with the amino acid residues at the active binding site of the target proteins.

### Ligand interactions of three phytocompounds with the highest drug scores

For SARS-CoV-2 M^pro^, the 2-dimensional interactions of the standard references (3WL and carmofur) and the phytocompounds (C_18, C_4, and C_5) with the amino acid residues at the active binding site is given in Fig. 4. The 3-dimensional biding pose of the phytocompounds and the standard references at the active binding pockets of SARS-CoV-2 M^pro^ is also given in Fig. 5. In general, the interacting amino acid residues of SARS-CoV-2 M^pro^ are GLN189, GLN192, MET165, PRO168, THR190, GLU166, ARG188, MET49, ASN142, HIS41, THR25, CYS44, GLY143 and THR26 (Fig. 4). The binding parameters of the standard references and the phytocompounds with the amino acid residues at the active binding site of SARS-CoV-2 M^pro^ are given in Table 5. The number of conventional hydrogen bonds formed by C_18 (*n* = 3), C_4 (*n* = 5), and C_5 (*n* = 4) with the amino acid residues at the active binding site of SARS-CoV-2 M^pro^ is more than the number of hydrogen bonds formed by the standard references ‘3WL’ (*n* = 2) and ‘carmofur’ (*n* = 2) (Fig. 4). Overall, C_18, C_4, and C_5 showed different types of interactions with 6, 7, and 4 amino acid residues at the active binding site of SARS-CoV-2 M^pro^ respectively. Among the interacting amino acid residues of the active binding site of SARS-CoV-2 M^pro^, GLN189 has the highest occurrence (*n* = 3) followed by ARG188 (*n* = 2), ASN142 (*n* = 2), MET49 (*n* = 2), GLU166 (*n* = 2), GLN192 (*n* = 2), THR190 (*n* = 1), THR25 (*n* = 1), HIS41 (*n* = 1), CYS44 (*n* = 1), THR26 (*n* = 1), GLY143 (*n* = 1), MET165 (*n* = 1), and PRO168 (*n* = 1).

For furin, the 2-dimensional interactions of the standard references (PGPRTRA and naphthofluorescein) and the phytocompounds (C_18, C_4, and C_5) with the amino acid residues at the active binding site is given in Fig. 6. The 3-dimensional biding pose of the phytocompounds and the standard references at the active binding pockets of furin is also given in Fig. 7. In general, the interacting amino acid residues of furin are ASP258, GLU299, ASN295, FMT601, SER368, HIS194, GLY229, LEU227, ASP191, THR365, ARG193, ARG197, ARG298 and HIS364 (Fig. 6). The binding parameters of the standard references and the phytocompounds with the active binding site amino acid residues of furin are given in Table 6. The number of conventional hydrogen bonds formed by C_18, C_4, C_5, PGPRTRA, and naphthofluorescein are 8, 3, 5, 7, and 2 respectively (Fig. 6). Overall, C_18, C_4, and C_5 showed different types of interactions with 6, 5, and 5 amino acid residues at the active binding site of furin respectively. Among the interacting amino acid residues of the active binding site of furin, FMT601 has the highest occurrence (*n* = 4) followed by ASN295 (*n* = 3), SER368 (*n* = 3), THR365 (*n* = 3), HIS194 (*n* = 2), LEU227 (*n* = 2), ARG298 (*n* = 2), ASP258 (*n* = 1), GLU299 (*n* = 1), GLY229 (*n* = 1), ASP191 (*n* = 1), ARG193 (*n* = 1), ARG197 (*n* = 1), and HIS364 (*n* = 1).

A hydrogen bond is an important facilitator for proper binding between a protein and a ligand [61]. In a similar fashion with the reference standards, the phytocompounds (C_18, C_4, and C_5) showed a good number of conventional hydrogen bonding with the amino acid residues at the active binding site of the target proteins (SARS-CoV-2 M^pro^ and furin) (Figs. 4 and 6). Hydrophobic interactions are noncovalent bonding interactions that are considered to be crucial for protein folding and protein–ligand interactions [62, 63]. Two phytocompounds (C_18 and C_5) showed hydrophobic interactions with the amino acid residues at the active binding site of SARS-CoV-2 M^pro^ (Fig. 4). Electrostatic interactions are also reported to be important for protein stability, function, flexibility, and folding [63]. Two phytocompounds (C_4 and C_5) showed electrostatic interactions with the amino acid residues at the active binding site of furin (Fig. 6). Analysis of ligand interactions revealed that all the three phytocompounds (C_18, C_4, and C_5) interacted with different amino acid residues at the active binding site of the target proteins (SARS-CoV-2 M^pro^ and furin).

### Pharmacological relevance of three phytocompounds with the highest drug scores in COVID-19 pandemic

Quercetin-3-*O*-*α*-L-rhamnopyranoside (C_18) has been reported to exhibit antiviral activity against the influenza A virus (H1N1) [64]. SARS-CoV-2 is a viral pathogen that primarily affects the respiratory system [65]. Pharmacological activities of C_18 like antioxidant, anti-obesity, and other activities have also been reported [66–68]. The antioxidant activity of C_18 might prove beneficial in SARS-CoV-2 infection as COVID-19 is associated with oxidative stress [65]. In addition to *A. pennata*, Quercetin-3-*O*-α-L-rhamnopyranoside is also found in other plants [64, 66–71]. Kaempferol 3-*O*-α-L-rhamnopyranosyl-(1 → 4)-β-D-glucopyranoside (C_4) and isovitexin (C_5) were reported to inhibit cyclooxygenase (COX)-1 and COX-2 enzyme in a COX-1/COX-2- catalyzed prostaglandin biosynthesis assay [32]. COX enzymes are of clinical relevance as they are pro-inflammatory agents that are inhibited by anti-inflammatory drugs [72]. The anti-inflammatory activity of C_5 against lipopolysaccharide-induced neuroinflammation has also been reported [73]. COVID-19 is associated with inflammation [74] and some studies suggest the treatment of COVID-19 by inhibition of COX-2 [75]. Moreover, the antioxidant activity of C_5 has also been reported [76]. In addition to *A. pennata*, isovitexin (C_5) had also been isolated from other plants [77–81]. The existing data of the phytocompounds suggests that they could be considered as potential leads. However, the bioavailability and the degree of difficulty to synthesize a compound must be evaluated for the promising phytocompounds before they could be considered as potential leads.

### Bioavailability score of the three phytocompounds with the highest drug scores

The process of drug development can be slowed down by bioavailability issues associated with a compound [52]. For a compound to be effective as a drug, it is important that a sufficient concentration of a compound is available within the systemic circulation for a specified period so that a compound can exert its pharmacological action on the body [51]. Just like toxicity, it is important to assess the bioavailability of a compound at an earlier stage in the drug development process to avoid unfruitful outcomes in the future. Therefore, the bioavailability of the phytocompounds (C_18, C_4, and C_5) was determined with the SwissADME web tool. The SwissADME web tool determines the bioavailability based on the molecular properties and lipophilicity of a compound by applying different principles such as Lipinski’s rule of 5, Ghose filter, Veber filter, Egan filter and Muegge filter [51]. Among the phytocompounds, C_5 has the highest bioavailability score (0.55) while C_4 and C_18 each have a low bioavailability score (0.17) (Table 7). The bioavailability study revealed that C_5 has better bioavailability than the other phytocompounds.

### Synthetic accessibility of the three phytocompounds with the highest drug scores

During the process of virtual screening to identify promising lead compounds, it is preferable to filter out a non-toxic, biologically active compound having good bioavailability [51]. Along with this, the degree of difficulty to synthesize a compound is also a factor that needs to be taken into consideration while selecting the most promising compound [51]. In our study, we have assessed the degree of difficulty to synthesize a compound with the SwissADME web tool. Synthetic accessibility is a fingerprint-based computational approach that can be used to determine the level of difficulty for synthesizing a compound [51]. A compound with a synthetic accessibility score of 1 indicates that the compound can be easily synthesized while a synthetic accessibility score of 10 suggests that the compound will be very difficult to synthesize [51]. Among the phytocompounds, C_5 (4.99) has the best synthetic accessibility score followed by C_18 (5.19) and C_4 (6.35) (Table 7). The synthetic accessibility study revealed that C_5 will be easier to be synthesized in comparison to the other phytocompounds.

### Structure–activity relationship of the most promising phytocompound (C_5) and comparison with other studies

Based on the binding affinity towards the target proteins, molecular properties, drug-likeness, toxicity, ligand interactions, bioavailability and synthetic accessibility, C_5 (Isovitexin) has been found as the most promising phytocompound that could act as a potential lead. Although C_5 showed good ligand interactions with SARS-CoV-2 M^pro^ and furin, it is important to make sure that C_5 interacted specifically with the active amino acid residues at the active binding site of the target proteins. Therefore, we decided to compare our experimental findings with several studies published by other researchers.

From Figs. 4, 6, and 8, it can be observed that the same functional group located at different positions on C_5 interacted with different amino acid residues at the active binding site of SARS-CoV-2 M^pro^ and furin. The structure–activity relationship of C_5 revealed that the –OH group at position 19 of the A-ring interacted with GLU166 of SARS-CoV-2 M^pro^ (Figs. 4 and 8) and also interacted with ASN295 of furin (Figs. 6 and 8). Several *in-silico* studies reported that many phytocompounds either formed conventional hydrogen bonds or interacted with GLU166 of SARS-CoV-2 M^pro^ [82–84]. An in-silico study also reported that several phytocompounds either formed conventional hydrogen bonds or interacted with ASN295 of furin [85]. The –OH group at position 28 of the 6-C glucoside attached to the A-ring of C_5 interacted with ARG188 of SARS-CoV-2 M^pro^ (Figs. 4 and 8) and also interacted with HIS364 and THR365 of furin (Figs. 6 and 8). Interestingly, other phytocompounds have also been reported to interact with ARG188 of SARS-CoV-2 M^pro^ [82, 86]. Also, several phytocompounds were reported to either form conventional hydrogen bonds or interact with HIS364 and THR365 of furin [85].

The –OH group at position 31 of the 6-C glucoside attached to the A-ring of C_5 interacted with GLN189 of SARS-CoV-2 M^pro^ (Figs. 4 and 8). Other compounds were also reported to either form conventional hydrogen bonds or interacted with GLN189 of SARS-CoV-2 M^pro^ [86, 87]. The oxygen atom at position 18 of the C-ring formed a conventional hydrogen bond with ASN295 of furin (Figs. 6 and 8). Several phytocompounds were also reported to either form a conventional hydrogen bond or interacted with ASN295 of furin [85]. The –OH group at position 27 of the 6-C glucoside attached to the A-ring formed a conventional hydrogen bond with THR365 (Figs. 6 and 8). Many phytocompounds either formed a conventional hydrogen bond or interacted with THR365 of furin [85]. The B-ring of the C_6_-C_3_-C_6_ scaffold of C_5 formed electrostatic interaction with ARG298 of furin (Figs. 6 and 8). Several phytocompounds were also reported to interact with ARG298 of furin [85]. It was observed that phytocompounds that interacted with the same active site residues of the target proteins similar to what we reported for isovitexin were published as potential inhibitors of SARS-CoV-2 M^pro^ and furin. After comparison with other studies, it was observed that C_5 has the potential to interact specifically with the active amino acid residues at the active binding site of SARS-CoV-2 M^pro^ and furin.

Isovitexin (C_5) is apigenin with a 6-C glucoside attached to position 3 (Fig. 8) of the A-ring [88]. Recently, a study showed that the methanol-trifluoroacetic acid leaf extract of *Vitis vinifera* was able to effectively inhibit the replication of SARS-CoV-2 in-vitro. From about 40 phenolic compounds present in the leaf extract of *V. vinifera*, isovitexin (C_5, apigenin-6-C glucoside) was among the most abundant phenolic compounds present in the leaf extract [89]. Although the study did not identify the phenolic compound responsible for the inhibition of SARS-CoV-2, the fact that isovitexin was among the major phytoconstituents present in the plant extract suggests that isovitexin might play an important role in the inhibition of SARS-CoV-2.

Interestingly, an *in-silico* study found apigenin as a potential inhibitor of SARS-CoV-2 M^pro^ [83]. A molecular docking simulation-based *in-silico* study found apigenin-o-7-glucuronide as a potential inhibitor of SARS-CoV-2 M^pro^. Molecular dynamics simulation reveal stable conformation between the SARS-CoV-2 M^pro^ and apigenin-o-7-glucuronide complex [84]. The in-silico findings reported by other researchers suggest a stable protein–ligand interaction between apigenin (with or without its sugar moiety) with SARS-CoV-2 M^pro^.

Pharmacophores could be any part of the structure of a compound that interacts with a target protein to exert a pharmacological activity [90]. The oxygen atom at position 18 of the C-ring, the –OH group at position 19 of the A-ring, the –OH group at position 27, 28, and 31 of the 6-C-glucoside attached to the A-ring, and the B-ring could be considered as the pharmacophores in the structure of isovitexin as they had interacted with the active amino acid residues at the active binding site of SARS-CoV-2 M^pro^ and furin (Figs. 4, 6, and 8). All these pharmacophores are attached to the basic flavonoid (C_6_–C_3_–C_6_) scaffold. Therefore, we conclude that compounds having oxygen atom at position 18 attached to the C-ring, –OH group at position 19 attached to the A-ring, and the presence of a 6-C-glucoside that is attached to the A-ring of a C_6_–C_3_–C_6_ scaffold (Fig. 8) could offer the best alternative to develop new leads against SARS-CoV-2.

## Conclusions

Since SARS-CoV-2 utilizes its main viral protease ‘M^pro^’ and human protease ‘furin’ to achieve cellular entry and viral replication, we have screened 29 phytocompounds reported to be isolated from *A. pennata* for potential leads using computational studies. Initially, our computational guided study revealed three flavonoids viz. quercetin-3-*O*-α-L-rhamnopyranoside, kaempferol 3-*O*-α-L-rhamnopyranosyl-(1 → 4)-β-D-glucopyranoside, and isovitexin as promising phytocompounds against SARS-CoV-2. However, quercetin-3-*O*-α-L-rhamnopyranoside and kaempferol 3-*O*-α-L-rhamnopyranosyl-(1 → 4)-β-D-glucopyranoside were not selected for further studies as they had bioavailability issues and were also found as difficult to synthesize. Based on binding affinity, molecular properties, drug-likeness, toxicity parameters, ligand interactions, bioavailability score, synthetic accessibility, structure–activity relationship and other supporting literary evidence, we found that isovitexin (apigenin-6-C-glucoside) was the most promising phytocompound to potentially inhibit the cellular entry and viral replication of SARS-CoV-2. Also, based on the structure–activity relationship, we conclude that compounds having oxygen atom at position 18 attached to the C-ring, –OH group at position 19 attached to the A-ring, and the presence of a 6-C-glucoside that is attached to the A-ring of a C_6_-C_3_-C_6_ scaffold could offer the best alternative to develop new leads against SARS-CoV-2. As the evidence provided in our study is limited to in-silico data only, further investigations such as in-vitro studies are suggested to understand the complete inhibitory potential of the phytocompound against SARS-CoV-2.

## Data Availability

Data and materials used in the study will be made available with proper request to the corresponding author.

## Abbreviations

COVID-19: Coronavirus disease 2019
SARS-CoV-2: Severe acute respiratory syndrome coronavirus 2
M^pro^: Main protease
SP: Spike protein
PGPRTRA: Para-guanidinomethyl-Phac-R-Tle-R-Amba
